# Public health consequences after ten years of the Syrian crisis: a literature review

**DOI:** 10.1186/s12992-021-00762-9

**Published:** 2021-09-19

**Authors:** M. H. D. Bahaa Aldin Alhaffar, Sandor Janos

**Affiliations:** 1 Damascus University, Damascus, Syria; 2 Head of the Department of Public Health and Epidemiology, University of Debrecen, School of Health Sciences, Debrecen, Hungary

**Keywords:** Syrian crisis, Syrian war, Healthcare system, Public health

## Abstract

Ten years of the Syrian war had a devastating effect on Syrian lives, including millions of refugees and displaced people, enormous destruction in the infrastructure, and the worst economic crisis Syria has ever faced. The health sector was hit hard by this war, up to 50% of the health facilities have been destroyed and up to 70% of the healthcare providers fled the country seeking safety, which increased the workload and mental pressure for the remaining medical staff. Five databases were searched and 438 articles were included according to the inclusion criteria, the articles were divided into categories according to the topic of the article.

Through this review, the current health status of the Syrian population living inside Syria, whether under governmental or opposition control, was reviewed, and also, the health status of the Syrian refugees was examined according to each host country. Public health indicators were used to summarize and categorize the information. This research reviewed mental health, children and maternal health, oral health, non-communicable diseases, infectious diseases, occupational health, and the effect of the COVID − 19 pandemic on the Syrian healthcare system. The results of the review are irritating, as still after ten years of war and millions of refugees there is an enormous need for healthcare services, and international organization has failed to respond to those needs. The review ended with the current and future challenges facing the healthcare system, and suggestions about rebuilding the healthcare system.

Through this review, the major consequences of the Syrian war on the health of the Syrian population have been reviewed and highlighted. Considerable challenges will face the future of health in Syria which require the collaboration of the health authorities to respond to the growing needs of the Syrian population. This article draws an overview about how the Syrian war affected health sector for Syrian population inside and outside Syria after ten years of war which makes it an important reference for future researchers to get the main highlight of the health sector during the Syrian crisis.

## Introduction

The Syrian war is without a doubt the largest humanitarian crisis of the twenty-first century. The war, which began in 2011, entered its tenth year by March 2021, with millions of displaced people both inside and outside Syria, and hundreds of thousands were slain, injured, disabled, or gone without a trace [1].

The movement which was a part of the Arab spring in 2011, turned into the biggest refugee’s crisis of the modern world, with millions of Syrian flee their houses and became refugees in other countries, and over six millions internally displaced, and enormous destruction to the infrastructure, healthcare system, social status of the population, economic crisis, and an increasing need for humanitarian support from the international community [2]. The international community failed to prevent the destruction of the health infrastructure, which resulted in the collapse of Syrian’s healthcare system and left millions of internally displaced people (IDPs) in desperate need of medical assistance [3].

Syria’s healthcare facilities and workers have been directly affected by the conflict and violence. These attacks have destroyed the public healthcare system, resulting in serious population health consequences such as an increase in infectious and non-communicable disease risks, serious maternal and child health challenges, conflict-related trauma, and mental health issues, as well as the exodus of Syrian healthcare workers who are seeking to flee the conflict [4]. Moreover, one of the most pressing concerns for Syrian refugees is access to health care. Primary health care and emergency lifesaving interventions are top priorities for UNHCR and its partners. The overall access to healthcare services is more difficult than any time before. Donors need to increase funding, personnel, and medical supplies to support these health needs until a diplomatic solution for Syria allows refugees to return safely [5, 6], as access to healthcare is one of the refugees’ rights [7].

Therefore, it is of great importance to document the health status of the Syrian population after ten years of war, to have an overview of the current health situation, the remaining health facilities, and the challenges facing the healthcare system. This review will benefit the future of the Syrian healthcare system as it highlights the significant gaps and suggests solutions to ensure equal access to healthcare for Syrian population, and to be prepared for the return of the refugees and their health needs in the future Syria.

### Aim of the research

This research aims to provide an overview of the current health status and health problems for the Syrian population.

## Methods

This scoping literature review covered the articles published on the subject of Syrian population health during the years of war. The modified Arksey and O’Malley framework for conducting the scoping review was used as the conceptual approach for the review process [8].

A combination of basic keywords and MeSH (medical subject headings) were used to identify relevant articles and publications through the different databases. The search strategy included the following: (“Syria” or “Syrian”) AND (“war” or “crisis” or “conflict”) AND (“Health”). The databases included in the search are the following: Medline Ovid, PubMed, Embase, Scopus, Google Scholar.

Articles were included if they covered any field related to the health of the Syrian population and Syrian refugees, the effect of the Syrian war on public health, healthcare for refugees, access to healthcare among Syrian refugees, and published after 2012. We excluded articles that are not related to the Syrian war, Syrian refugees, articles on general medical subjects not related to the Syrian crisis, articles on animal research, articles published before 2012, articles not written in English, and gray literature. Articles were included with or without the availability of full text.

We included primary research, secondary research, and different study designs (cross-sectional, cohort, cases control, literature review, systematic review), and excluded news, editorials, letter to the editor, reports, and conference proceedings.

After applying the research strategy on each database, results were imported into Endnote database, first, the duplication was automatically removed, and then the titles and the abstracts were screened independently by two teams (each team has two reviewers) to remove articles not related to the health of the Syrian population during the years of war. Articles were divided into different categories (e.g. mental health, oral health, etc..) according to the World Health Organization (WHO) categorization of health areas [9], and any disagreement between the reviewers was solved by discussion or consulting a third reviewer. PRISMA 2020 flow diagram was used to present the results of the literature search, and to identify the included and the excluded articles with the reason of exclusion [10].

### Results of the search

**Fig. 1:**
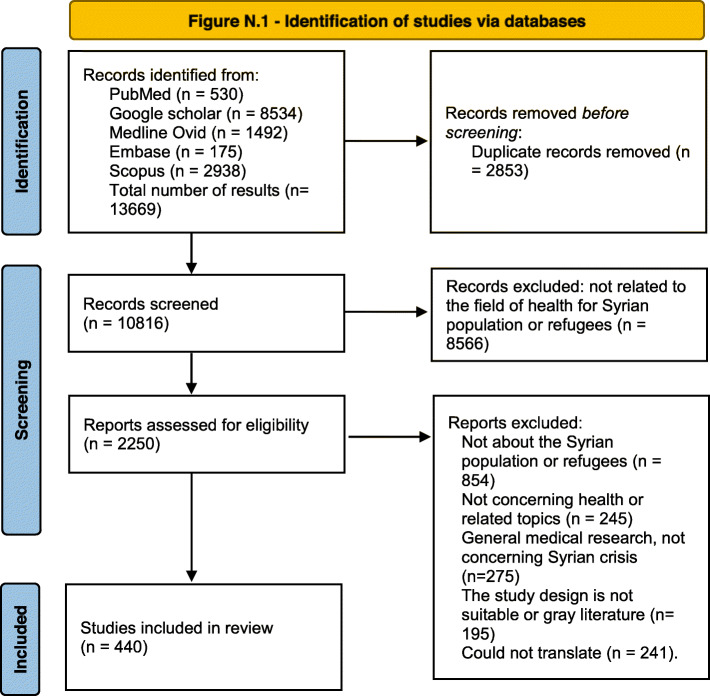
Identification of studies via databases

Figure 1 represents the search strategy and the citations included and excluded through the process. Five databases were screened following the search strategy described in the methods section, a total number of (13669) references appeared through the search, 530 in PubMed, 8534 in Google Scholar, 1492 in Medline Ovid, 175 in Embase, and 2938 in Scopus. References were imported into Endnote citation manager library, an initial check was made for the search results, and 2853 were removed as a duplicated article. Total number 10816 were included in the initial screening and 8566 were excluded as they were not related to the research topic.

2250 were screened through abstract and full text (if available), and references were excluded for the following reasons:

Not about the Syrian population or refugees (*n* = 854).

Not concerning public health or related topics (*n* = 245).

General medical research, not concerning Syrian crisis (*n* = 275).

The study design is not suitable or gray literature (*n* = 195).

Could not translate (*n* = 243).

The final number of references included in this literature review was 438, references were categorized into the following categories: mental health, children and maternal health, non-communicable diseases, infectious diseases, oral health, access to health care for the population inside Syria, access to healthcare for Syrian refugees, occupational health, health systems, health during COVID-19 pandemic.

### Current health status for the population inside Syria

Syria’s healthcare system has been severely damaged, and a large number of physicians and healthcare providers have fled the country during the conflict, which increased the pressure and the workload on the remaining healthcare facilities and healthcare providers.

Health facilities in the areas under government control slowly lost the ability to function and provide healthcare services for the Syrian population. The destruction of major health facilities and drug factories, the increased number of doctors leaving the country, and the movement of millions of internally displaced people toward safe cities were the main reasons for the low function of the remaining health facilities [11]. On the other hand, many attempts have been established to create a primary healthcare system in the most vulnerable sides of Syria, which is mainly the opposition territories. Those attempts have made an enormous effort to provide access to a variety of basic healthcare services, including primary care. Despite these efforts, the current system remains fragile and unsustainable [12]. However, relatively few papers on the current state of Syria’s healthcare system have been published. Most articles do not discuss the Syrian healthcare system’s capabilities; however, the information available points to massive humanitarian and health needs for the Syrian people, which necessitates international engagement to offer the necessary assistance.

#### Health status for the Syrian refugees

Starting from 2012, the number of Syrian refugees has increased dramatically, people fled across land and sea to reach safety in the neighboring countries [13], by the end of 2020, statistics showed that the number of Syrian refugees outside Syria exceeded 6 million, the majority of them lives in Turkey which is the host of 3.6 million Syrians, Lebanon which host almost a million, and Jordan which hosts over 600 thousand, and more hundreds of thousands in Iraq and Egypt [14]. According to the European migration counsel, 1.3 million Syrians requested asylum in Europe, and the peak of the migration was in 2015–2016, and it had declined significantly since then. Most of the refugees have limited access to basic services [15, 16].

Turkey has had an open-door policy for refugees since the beginning of the Syrian refugee crisis, with over 3 million refugees entering the country and about 220,000 people living in camps [17]. This explosive and unexpected increase in the Syrian population in Turkey has had several negative impacts on health and social determinants. Turkey has 20 large refugee camps spread across ten cities. The Ministry of Health runs 21 field hospitals within the camps, with 120 doctors and 400 allied health personnel working there. Also, 25 Syrian doctors work in clinics run by nongovernmental organizations (NGOs) and in refugees camps, providing medical care to fellow refugees [18]. The rate of incidence of preventable diseases has increased in Turkish residents as a result of lower vaccination rates for polio and measles. Furthermore, because the rate of tuberculosis has increased in Turkey in 2014, refugees, particularly those living outside of camps, pose a significant health risk. In large cities like Istanbul, Ankara, and Izmir, many refugees live in deplorable conditions and are homeless [18, 19]. Furthermore, communicable diseases pose a serious public health threat to both refugees and residents of the host country. Several factors increase the risk of refugees contracting communicable diseases. The high prevalence of infectious diseases in the country of origin, as well as exposure to new communicable diseases in transit and host countries, are among these factors. Inadequate food, water, and sewage, as well as incomplete immunization, ecological change, contact with novel antigens, crowded and unsanitary living conditions, and lack of access to adequate food, water, and sewage, all increase the risk of infectious diseases. Reproductive health services necessitate special consideration [20].

Lebanon, which is the closest country to Syria (about one hour drive from Damascus) hosts a very high percentage of Syrian refugees taking into account the Lebanese population which is around 4 million [21]. Estimation reports that the number of Syrian refugees in Lebanon is around 1.3 million [22]. Tuberculosis, leishmaniasis, hepatitis A, and measles have become more common among refugees. Lebanese doctors are unfamiliar with Leishmaniasis treatment and been struggling to keep up with the rising number of cases. Furthermore, chronic diseases such as type 2 diabetes, cardiovascular disease, hypertension, chronic obstructive pulmonary disease, musculoskeletal pain, and surprisingly epilepsy, are prevalent among refugees [23]. However, due to the current funding situation, few resources are available to adequately treat chronic conditions or provide antenatal and postnatal care. There are no services or treatments available for cancer patients. Moreover, Lebanese doctors reported outbreaks of cholera, typhoid, and hepatitis A, among those refugees living in informal urban and rural settings [24]. The current political and economic crisis in Lebanon has further worsened the health situation for the refugees, the major factors are the lack of international support and the hard access to health care facilities which also triggered outbreaks of treatable diseases among the refugees [21, 22, 24]. The healthcare system in Lebanon is faced by the rapid increase of its population by 30% as a result of the massive influx of refugees, who can’t gain access to health care for refugees [23]. Therefore, the majority of the refugees are left for their fate, and the small acts of non-governmental organizations, and informal healthcare workers [25, 26]. Now communicable diseases, women’s health, and mental health are the main health problems of Syrian refugees in Lebanon [27].

In Jordan, the health situation of the Syrian refugees is different, as over 70% of the refugees are residing among host Jordanian communities, and only 30% are living in camps. The largest camp in Jordan is called Zaatari with an estimated population of 120,000, and for those, not all needs are addressed as they are not allowed to exit the camp and access the health facilities [21, 28]. Acute and communicable diseases, chronic diseases, and dental problems were all prevalent. Advanced services were more difficult to obtain than preventive and primary health care. Access was hampered by structural and financial barriers. Primary health care for adults and children with acute illnesses was the most common service, with more than half of the refugees requiring it, followed by vaccination and dental services. Over a third of the refugees said they needed primary health care for chronic illnesses [29]. Syrian refugees in Jordan’s non-camp settings have difficulty accessing health services, primarily due to financial situations. The transition from free to subsidized health services, as well as the gradual deterioration of economic status that occurs in many refugee households as a result of prolonged displacement, are likely to exacerbate this barrier. The Jordanian healthcare system has been overburdened as a result of refugees’ reliance on the public sector for primary and specialist care. Increased co-pays for public services, as well as a shift toward private-sector services, are likely to reduce refugee access to services [30].

In Europe, the economic and social costs of absorbing large numbers of refugees have alarmed European countries. They are doubting their ability to provide more humanitarian aid. Serbia and Germany account for the majority of Syrian refugees in Europe (57%) compared to (31%) in Sweden, Hungary, Austria, the Netherlands, and Bulgaria, and (12%) in the remaining 37 European countries. Health officials fear that the influx of refugees into Europe will introduce infectious diseases that have historically had low rates of morbidity and mortality in the host countries. Among these diseases are measles, polio, hepatitis A, hepatitis B, tuberculosis, human immunodeficiency virus (HIV), hepatitis C virus (HCV), cutaneous leishmaniasis, schistosomiasis, and MERS-CoV. This is largely due to the collapse of Syria’s healthcare infrastructure, which resulted in the suspension of the country’s vaccination programs [16]. Psychiatric disorders and unspecified somatic symptoms were surprising of high number among the young age group of refugees [31]. Barriers to quality health care for both physical and mental health problems are frequently cited as language and translation issues. For those with limited language skills, interactions with health care professionals can be intimidating, from discussing medical history to describing the characteristics and duration of symptoms. Syrian refugees in Germany face all of these challenges. They were made worse by the large number of people who arrived in a short period, making it difficult to practice the new language [32]. The European response to the refugee crisis was an emergency response that needed more structural changes in the EU healthcare system [33, 34].

The major number of Syrian refugees are settled in Turkey, Lebanon, Jordan, and Germany. More countries have hosted the Syrian refugees such as Iraq which hosts about 250 thousand, Egypt 150 thousand approximately, and Canada which hosts over 50 thousand, and many other European countries have hosted thousands of Syrian refugees for years. Each of the previous countries has its unique healthcare system and allowed different levels of access to health care services for the refugees.

### Public health indicators of the Syrian population

#### Maternal and children health

Children and maternal health are considered one of the most important public health aspects of any population, especially after war or disaster, as they are the worst affected by the war and the most vulnerable. The number of children affected by the Syrian war is shocking, reports until the end of 2014 state that over 12,000 children had been killed during the war, with no accurate reports after 2014 on the true number of the loss in children’s lives. Moreover, by 2015, 5.6 million children needed assistance, 3.8 million children were internally displaced and a further 2.1 million children were refugees in nearby countries [35]. Younger children were more likely to have an incomplete vaccination status [36]. In 2017, data collected from Northwestern Syria reported that respiratory diseases were the most commonly encountered illnesses across all age groups (27%), except for late teen females, who had the most gynecological/obstetric complaints. Across all age groups, infectious diseases caused the most disease burden, with upper respiratory tract infections (URTIs), infectious diarrhea, and otitis media accounting for nearly half (47%) of all cases. Nutritional deficiencies were discovered in 8% of the patients, the majority of whom were infants and toddlers (92%). Acute diarrhea was identified in 17% of all age groups, making it the second most common condition after URTIs [37].

Women’s health suffers disproportionately during times of conflict. Sexual and gender-based violence, a reduction in the use of modern contraceptives, menstrual irregularity, unintended pregnancies, preterm birth, and infant morbidity are issues that persist in all settings. Taking a multilevel approach to eliminate social and service delivery barriers that prevent access to care, conducting thorough needs assessments, and developing policy and programmatic solutions that establish long-term care for Syrian refugee women are among the recommendations for improved practice [38]. In refugees’ camps in Lebanon and Jordan, almost all births took place in a health facility (98% in Jordan and 94% in Lebanon). Cesarean delivery rates were similar in both countries, accounting for roughly one-third of all births. Exposure to war-related events was linked to maternal post-traumatic stress (PTS) and general psychological distress both directly and indirectly through daily stressors. Negative parenting and child psychosocial difficulties were directly linked to mothers’ general psychological distress, but not PTS, which can cause an increase in the risk of negative parenting behavior [39]. For the population inside Syria, 24% of pregnant women are adolescents due to the increase in early marriage, and the main problem is the lack of access to antenatal (ANC) and postnatal (PNC) healthcare. Statistics found that 85, 82, 44% of the pregnant women did not have a single ANC visit in the first, second, and third trimmest retrospectively. The current situation can be briefly described as a significant lack of ANC and PNC visits, a high adolescent birth rate, and a higher cesarean-to-vaginal delivery ratio than what the WHO recommends [40, 41].

#### Mental health

There has been a considerable number of publications related to the mental and psychological health and status of the Syrian population and Syrian refugees, both inside (IDPs) and outside Syria. Those articles discussed almost every factor affecting mental health, and most of the articles used the most recent diagnosis methods available. The high prevalence of post-traumatic stress disorder (PTSD) was between 36 and 61% in 2013 of the population in Syria, exposure to fighting and hostility, and a history of trauma during the current conflict were the main predictors of current symptoms of PTSD [42, 43]. Moreover, some studies focused on specific groups like the survivors of torture, because very limited data is available on the mental health of this specific group. Survivors of torture are more likely to develop psychological issues, such as depression, posttraumatic stress disorder, panic attacks, chronic pain, medically unexplained somatic symptoms, and suicidal behavior [43]. In a nationwide study conducted in 2020 and included about 2000 participants from different Syrian governates, 44% of Syrian participants living inside Syria are more likely to have a severe mental disorder, 27% had both likely severe mental disorder and full PTSD symptoms, 36.9% had full PTSD symptoms, and only 10.8% had neither positive PTSD symptoms nor mental disorder on the K10- scale. Even years after the traumatic event, 86.6% of respondents believed that the war was the main cause of their mental distress [44, 45]. The conflict in Syria has put the inside population at a higher risk of mental illness than Syrian refugees elsewhere. Many measures, with a focus on mental health, are required to aid people in avoiding long-term suffering [46–48].

#### Oral health

Few articles have addressed the state of oral health during the Syrian crisis, as well as its impact on Syrian refugees. A high prevalence of caries, periodontal diseases, and periodontitis were noticed among the refugees because oral health and dental treatments are not considered a priority for the refugee’s overall health. Dental caries and odontogenic infections have increased, including acute periapical abscesses and even orofacial infections [49]. The average number of DMFT (Decayed, Missed, or Filled tooth which is an oral health index based on the total number of decayed, missed, or filled teeth) among all children was 3.36, which is higher than the WHO-recommended number. Only 14% of the sample in the study had good oral health, while 86% had at least one decayed, missing, or filled tooth. There was also a strong link between a child’s socioeconomic status and his or her oral health [50, 51].

#### Non-communicable diseases

Despite the high importance of providing health services for NCDs, very limited research articles and resources have been found covering this issue [52]. The World health organization (WHO) published a report in 2016 regarding the prevalence of NCDs for the population living inside Syria. Cardiovascular diseases had the highest percentage of NCDs with almost 25% of all the cases, 9% were cancer, 2% chronic respiratory diseases, 1% diabetes, 5% had communicable maternal or perinatal and nutritional conditions, and 8% for other NCDs, while war-related injures had a significantly high percentage for about 50% [53]. In 2016, cross-sectional research studied the prevalence of non-communicable diseases in Lebanon among Syrian refugees and compared the results to the host community. Over half of the Syrian refugees reported at least one of the five main NCDs which are (hypertension, cardiovascular disease, diabetes, chronic respiratory diseases, and arthritis) [54]. Among the refugees, arthritis had the highest prevalence (60%), followed by hypertension (47%), chronic respiratory diseases (38%), cardiovascular disease (3.3%), and diabetes (3.3%) [55].

#### Infectious and communicable diseases

The Syrian war has created the ideal conditions for the spread and outbreak of infectious and treatable diseases, the interruption in the vaccination program, the destruction of the health infrastructure, the migration of healthcare workers, along with the mass movement of the refugees to other countries and living without the proper health conditions in refugees camps where significant reasons for the epidemics of infections such as tuberculosis, leishmaniasis, polio, measles, hepatitis, and other infectious diseases, among both the refugees and within the hosting communities [56]. Since poliomyelitis thrives in unsanitary, crowded conditions and among malnourished children, it has been declared a public health emergency in Syria, requiring international efforts and solidarity to prevent a global epidemic. The WHO estimates that over 7600 Syrians are currently infected [57–59]. High Tuberculosis (TB) rates were found among Syrian refugees in Jordan through active screening and will probably persist as the Syrian crisis continues [60]. In Lebanon, an increasing number of TB cases have been reported among refugees. Since 2011, the number of TB cases in Lebanon has increased by 27%, and Syrians were responsible for 22% of the estimated TB cases in Jordan in 2013 [61, 62]. Other infections have been reported, including acute diarrhea and hepatitis A., Malaria outbreaks appear to be unlikely, as Syria had eradicated the disease before the conflict. A Plasmodium vivax outbreak imported from Iraq in 2012 resulted in 291 cases. In Syria, as well as among Syrian refugees in Lebanon, there has been an increase in the number of typhoid fever cases. In Iraq, there has been a cholera outbreak among Syrian refugees [61]. Another hidden consequence of the Syrian crisis is the rise of antibiotic-resistant bacteria. As a result of mis-prescribing and overprescribing antibiotics, the antibiotic resistance phenomenon is widespread in Syria, with a high rate of multidrug resistance cases in both Gram-negative and Gram-positive organisms during and after the Syrian crisis [63, 64].

#### Occupational health and healthcare providers situation

Estimation found that up to 70% of healthcare workers have fled the country, seeking a better situation or surviving the attacks on healthcare facilities during the years of war [65]. Moreover, less than 64% of hospitals and 52% of primary healthcare facilities in Syria are operational. According to the United Nations, this fact put greater pressure on the remain active healthcare workers inside Syria [66–68]. The international organization has tried to protect the healthcare workers on both sides of the Syrian conflict; however, those attempts have failed to achieve any significant protection for health workers and facilities. In late 2013, Al-Kindy Hospital, which is the largest hospital in the Middle East, and specialized in treating cancer patients, has been destroyed, this case is among many more revealing the true losses of the health sector in Syria [69–71]. Psychological distress among healthcare providers in Syria has been documented in several studies. In 2017, high levels of depression, anxiety, and stress were noticed and the percentage were 60.6, 35.1, and 52.6%, respectively [72]. In 2019, the consequences of the war on healthcare providers got even worse. The research studied the prevalence of burnout syndrome among the healthcare providers in Syria found that 93.75% of the residents had a high level of burnout in at least one of the three domains of the burnout index (emotional exhaustion, depersonalization, personal accomplishment), and 19.3% had a high level of burnout in all three domains. This high prevalence of burnout syndrome highlights the role of the current situation in raising the workload on the Syrian residents and healthcare providers [73]. Furthermore, despite all the challenges facing healthcare providers which are the high load of work, the increased psychological distress, and working in low-resources settings with very limited equipment, healthcare providers in Syria are facing an increased rate of workplace violence which further impacts the psychological status of healthcare workers. 84% said they had been exposed to workplace violence in the 12 months leading up to the survey. 74% were exposed to verbal violence, while 19% were exposed to physical violence. There was a significant positive correlation between verbal and physical violence and each item of depression and stress, as well as a significant negative correlation between subjective sleep quality and subjective health [74].

#### Healthcare system in Syria during COVID-19 pandemic

The Syrian government’s response to the pandemic started with the complete closure of the country by the end of March. The health quarantine lasted for 2 months but had a deep impact on the economic situation in the country. Therefore, the restrictions were canceled by June, which led to an increased number of cases and a significant rise in the death rate among the population [75, 76]. A recent study reported the perspective of the internally displaced population towards the COVID-19 pandemic. Household crowding, inadequate sewerage, and waste management, insufficient and poor-quality water, and a lack of cleaning supplies are among the issues that the Syrian population described as impeding an effective COVID-19 response. Participants cited the internet as their primary source of COVID-19 information, followed by NGO awareness campaigns. COVID-19 data was more accurate and comprehensive for men than it was for women. Although it did not appear to be possible to isolate high-risk people within households, participants suggested that ‘house-swapping approaches might work”. While the majority of participants knew enough about COVID-19 to prevent transmission, they lacked the practical tools to do so [77, 78]. Another cross-sectional study, which assessed the knowledge towards COVID-19 pandemic among the Syrian residents showed that the general knowledge about the pandemic was about 60%, and this percentage significantly differ between the age groups, gender, and educational level [79]. Another important study published on the impact of COVID-19 on the healthcare providers in Syria concluded that healthcare providers during the pandemic suffered from poor sleep quality, severe stress disorder, and high rates of anxiety. The pandemic further increased the workload over the remaining health workers inside Syria [80].

##### Current and future challenges facing the Syrian healthcare system

It is clear now that the amount of destruction in the Syrian healthcare system is enormous, and the health needs and consequences of the war are growing day by day. Moreover, no single organization, government, or health authority can do anything on its own to provide healthcare and to respond to the health needs of the Syrian people [81]. Therefore, rebuilding the Syrian healthcare system is complicated as many factors play a major role in how effective the health system can be, such as the capacity of the institutions and the capacity of individuals, the economic set-up, the political context, and the ability to adopt new approaches to governance and financing [82].

Essential steps should be adopted in any future strategy to build the healthcare system include reparation of the health infrastructure, formation of a high-level task force involving policymakers and other stakeholders, training of the human resources, developing tools for assessments of the healthcare system, support the medical research [83]. Previous steps along with many others including enhancing the capacity of local organizations [84], improving the interaction between the stakeholders, adoption of lessons from other countries went through similar changes [34], and increase the capacity of health-related research [85] can be a great opportunity and a turning point for the Syrian healthcare system, to enabling equal access to healthcare services for the population, and ensuring complete health coverage despite the geographical location and the social class [86].

Major challenges are facing the future Syrian healthcare system, one major concern is regarding the return of the Syrian refugees from the neighboring countries and their need to access healthcare services which will be overwhelming for the healthcare system [17], and this raises the question about the current situation of the refuges which is different in each country [87], the future capacity of the Syrian healthcare system, and the steps to be done to increase this capacity. Mental health is also one of the major challenges in the future, the medical literature showed a high prevalence of mental disorders due to the war, however, limited interventions have been made in this field [88], and future research should focus on proposing intervention which helps the people with mental disorders. Oral health is considered a secondary health issue, however, in the future, there will be an increased demand for oral health, more research needs to be done in this field to understand the need and present solutions to improve oral health [89]. Regarding NCDs, there are no accurate statistics about the prevalence, morbidity, and mortality of the NCDs especially for the population inside Syria, therefore, more cross-sectional studies are needed to understand the burden of those diseases among the Syrian population which will help in the future health plans [52]. On the other hand, the previous spread of treatable infectious diseases in the hosting countries is an indicator that those infectious diseases might spread again inside Syria without proper management and vaccination plans after the returning of the refugees, more research is needed in the field of infectious diseases and microbial resistance among refuges to have a deeper understanding and avoid possible spread of diseases in the future [64, 90].

## Conclusions

The amount of destruction that the Syrian war has left behind after ten years is indescribable, and the humanitarian support from the international community is not equal to the growing needs of the population.

Healthcare services in the opposition-controlled areas are limited and hard to access, some attempts to rebuild the healthcare system have already begun, however, no significant change has been made until now. For the refugees in the neighboring countries, access to healthcare services is different according to the host country, financial problems, limited access to healthcare, and the outbreaks of many diseases were the major problems.

Future health research concerning the Syrian population should focus on effective intervention to treat mental health problems, increase the capacity and access to maternal health, more focus on oral health, preventive measurement against NCDs, and the prevention of outbreaks and the spread of infectious diseases after the return of the Syrian refugees.

In conclusion, the Syrian war had many consequences over the public health aspects, which extended the Syrian population to affect the hosting communities. The Syrian healthcare system cannot be rebuilt without the collaboration of all the authorities engaged in the Syrian conflict, with the help of international organizations and health institutes to reestablish the missing health services and provide universal healthcare access and equality for the Syrian population. This review can help future researchers to understand the different aspects of health during the Syrian crisis as it provides an overview of the changes and problems related to the health sector during and after ten years of the Syrian war.

### Limitations of the study

The authors of this literature review tried to summarize the available published articles to cover the public health consequences related to the Syrian crisis. However, due to the limited number of articles with original data, a systematic review with meta-analysis was not able to be conducted, which could be of higher medical evidence.

## Data Availability

The datasets used and/or analyzed during the current study are available from the corresponding author on reasonable request.
